# Pathologic Mechanisms of the Newcastle Disease Virus

**DOI:** 10.3390/v15040864

**Published:** 2023-03-28

**Authors:** Di Zhang, Zhuang Ding, Xiaohong Xu

**Affiliations:** Key Laboratory of Zoonosis Research, Ministry of Education, Jilin University, Changchun 130062, China

**Keywords:** Newcastle disease virus, pathologic mechanism, cytokine, receptor, autophagy, apoptosis

## Abstract

Newcastle disease (ND) has been a consistent risk factor to the poultry industry worldwide. Its pathogen, Newcastle disease virus (NDV), is also a promising antitumor treatment candidate. The pathogenic mechanism has intrigued the great curiosity of researchers, and advances in the last two decades have been summarized in this paper. The NDV’s pathogenic ability is highly related to the basic protein structure of the virus, which is described in the Introduction of this review. The overall clinical signs and recent findings pertaining to NDV-related lymph tissue damage are then described. Given the involvement of cytokines in the overall virulence of NDV, cytokines, particularly IL6 and IFN expressed during infection, are reviewed. On the other hand, the host also has its way of antagonizing the virus, which starts with the detection of the pathogen. Thus, advances in NDV’s physiological cell mechanism and the subsequent IFN response, autophagy, and apoptosis are summarized to provide a whole picture of the NDV infection process.

## 1. Introduction

*Avian paramyxoviruses* 1(APMV-1) is commonly known as Newcastle disease virus (NDV). The virulent strain can cause Newcastle disease (ND), which is a highly contagious and acute disease in avian species worldwide. The strain causes severe economic losses in the poultry industry, and it was first identified in Newcastle of England and in Indonesia in 1926. Recently, it was categorized as *Avian orthoavulavirus* 1 of the *Orthoavulavirus* genus, *Avulavirinae* subfamily and *Paramyxoviridae* family according to the updated unified phylogenetic classification system and revised nomenclature for Newcastle disease virus. The genome of NDV is a single-stranded, negative-sense RNA encoding eight gene products, namely the nucleoprotein (NP), phosphoprotein (P), matrix (M), fusion (F), hemagglutinin-neuraminidase (HN), the RNApolymerase (L), and V and W proteins [1]. The length of the NDV genome consists of multiple of six nucleotides (nt): either 15,186, 15,192, or 15,198 nt [2].

The components of the virus work together to accomplish the entire invasion and infection process. All proteins have their unique functions and cooperate with each other. With respect to the nucleoprotein, NP is responsible for the stable encapsidation of the NDV genome, and it is assisted by the P and L proteins [3]. The P protein is engaged in viral RNA synthesis and enables the solubility of the NP [4]. The M protein is the skeleton of the virus and is essential for viral budding [5]. It also promotes viral replication by regulating charged multivesicular body protein 4 [6]. HN and F proteins are key factors for the virus to enter and release from host cells. The F protein is primarily involved in virus entry, cell fusion, and hemolysis [7]. It is synthesized as a precursor protein, F0. It can mediate virus–cell membrane fusion after cleavage into F1 and F2 polypeptides. The cleavage is performed by host cell proteases, and the sensitivity of diverse strains of F proteins to different proteases is determined by the fact that F proteins have differentamino acid motifs at the cleavage site [8]. The cleavage site molecular basis of the F protein (FCS) is most decisive with respect to the pathogenicity of NDV strains. The FCS of virulent and mesogenic strains is 112R/K-R-Q-R/K-R F117, while for lentogenic strains, the FCS is 112G/E-K/R-Q-G/E-R L117 [9]. Additionally, the specific amino acid sequence of the F protein is deeply associated with the tropism relative to the brain, lung, and spleen tissues [10]. The L protein is the largest protein in the virus, and it is supposed to be multifunctional. It is involved in genome replication and in the transcription and regulation of viral replication in cells [11,12]. The W and V proteins are nonstructural proteins generated from the RNA editing process of the P gene. The V protein is critical for the inhibition of host type I interferon (IFN) secretion and apoptosis, providing a favorable environment for viral replication [13,14,15]. The W protein is the other RNA editing product of the P gene, which could be expressed in the nucleus or the cytoplasm depending on the genotype of the viral strain [16].

The epidemiology, immune response against NDV infection, and the evolution of NDV strains have been extensively and concisely reviewed [17,18,19,20,21,22,23,24]. However, the pathogenic infection mechanism has not yet been summarized. To provide a better understanding of NDV infections, apoptosis, and IFN-related mechanisms, the host cell responses against NDV infection are described in detail in this review.

## 2. Clinical Signs and Damage to Organs

The virulence of NDV is determined by multiple factors, including tissue or organ tropism, ability to resist the host’s immune system, and replication efficacy. The mean death time (MDT) in embryonated chicken eggs, the intravenous pathogenicity index (IVPI) in 6-week-old chickens, and the intracerebral pathogenicity index (ICPI) in 1-day-old chickens are indicators for assessing NDV virulence in vivo [25]. NDV strains with an ICPI value below 0.5 are considered lentogenic, while strains with ICPI values of 0.5–1.5 and 1.5–2.0 are classified as mesogenic and velogenic, respectively [26]. The clinical manifestations of NDV differ depending on its virulence. Lentogenic NDV strains cause subclinical infection with mild respiratory or enteric disease and are considered low-virulent. Mesogenic NDV strains have intermediate virulence and cause respiratory infections with a moderate mortality rate. Velogenic strains are divided into two types: viscerotropic velogenic strains and neurotropic velogenic strains. The former cause ulcerative hemorrhages in the gastrointestinal tract; lymphoid depletion; and necrotic foci in the spleen, liver, and gut-associated lymphoid tissue (GALT). Whereas neurotropic velogenic strains are characterized by dyspnea, depression, opisthotonos, head twisting, and paralysis [27]. NDV infections also perturb the ceca flora of chickens, displayed as an increased abundance of pathogenic *Rhodoplanes* and *Clostridium*, along with the depletion of benign bacteria such as *Paenibacillus* and *Enterococcus* [28].

NDV strains vary greatly in terms of tissue tropisms and severity of clinical symptoms. For example, during infection with Goose/CH/GD/E115/2017 (E115) (ICPI of 1.67), a chicken flock displayed neurological signs, including head twitching, muscle tremors, and even paralysis and diarrhea, whereas infected geese did not show any clinical symptoms. The presence of the virus has been detected in the lungs, spleen, kidneys, trachea, bursal sac, glandular stomach, cecum, and liver of infected chickens and geese; however, it is worth noting that the virus is present in the brain of chickens and not in geese [29]. NDV can also cause damage to pancreatic tissue in chickens, manifested by the reduced production and activity of pancreatic digestive enzymes, as well as increased corticosterone and somatostatin levels and decreased insulin production. Pancreatic damage caused by NDV may lead to a decline in growth performance [30]. Another study investigated an outbreak of Newcastle disease in wild pigeons in São Paulo, Brazil, in 2019. Infected birds showed neurological signs, and bleeding was observed in different tissues. Histopathological changes with the infiltration of monocytes were also found in the brain, kidneys, ventricles, heart, and spleen [31].

The cross-species transmission of the Newcastle disease virus has also been reported in recent years. Chickens exposed to pigeons with velogenic viscerotropic Newcastle disease—genotype VIId experienced higher mortality and severe respiratory, digestive, and neurological signs and higher and longer virus expulsion times in the cloaca than in the oropharynx. Diseased Muscovy ducks can cause chickens that have been in contact with them to develop obvious NDV symptoms. Specific manifestations include conjunctivitis, swelling of the head, respiratory signs of sneezing and a runny nose, and neurological signs such as tremors and torticollis [32,33]. This proves that pigeons and Muscovy ducks are considered effective carriers of the AOAV-1 genotype VIId virus and are risk factors for Newcastle disease virus infections in commercial chickens, thereby reducing productivity. Experiments with five-week-old chickens infected with pigeon-derived NDV with genotype XXI showed that chickens could be infected with the virus and exhibit high mortality and typical symptoms of the Newcastle disease. There were obvious pathological changes mainly in the lungs, thymus, spleen, and bursa of chickens; in addition, obvious neuronal lesions were also found in the brain of infected chickens, including glial hyperplasia and neuronal degeneration [34]. The serial passage of pigeon-derived NDV in chickens reveals an increase in pathogenicity in chickens, which also raises the possibility that wild bird populations should be taken into account when monitoring NDV.

The clinical symptoms of NDV are not very characteristic and are easily confused with highly pathogenic avian influenza, infectious bronchitis, infectious laryngotracheitis, fowl cholera, mycoplasmosis, and psittacosis [35]. However, what is common is the lymphoid tropism of virulent strains. Virulent strains lead to lymphoid depletion in bursal and thymic tissues and severe apoptosis in the spleens of chickens [36,37]. Yang reported that NDV strain F48E9 (ICPI of 1.93) displayed atrophy relative to the bursa of Fabricius (BF) with severe damage, and genes associated with the innate immune response were significantly upregulated [38]. Lu et al. showed that a duck-origin NDV strain induced obvious histological lesions of the lymphoid tissues, including lymphoid necrosis and lymphoid depletion in the spleen, thymus, and BF. The NDV load in these organs was also correlated with the severity of clinical symptoms and damage relative to immune tissues [39]. CD3^+^ and CD4^+^ T lymphocyte populations were also proven to decrease in the spleens of chickens after infection with NDV AF2240 (ICPI of 1.9) and IBS002 (ICPI of 1.76) strains. However, KUL01^+^ macrophages significantly increased by more than 10-fold [40]. NDV inoculation also decreased CD25^+^ intraepithelial lymphocytes, especially in the case of virulent strains (GVII and GVIII) [41].

## 3. Cytokine Secretion during NDV Replication

NDV has long been considered to be a strong stimulator of inflammatory cytokines, especially IFNs. It is highly related to the pathogenesis of NDV. The inducibility of IFN is highly variable depending on different strains and host types. Most strains do not tend to induce IFN production in chickens. Anis et al. suggested that weaker IFN-β expression was detected in the lung tissues of chickens than in ducks infected with the lentogenic 9a5b NDV strain, which may be associated with clinical severity [36,42]. Similarly, IFNs were not upregulated in the BF of chickens or in CEFs when challenged with the velogenic NDV strain F48E9 (genotype IX), although several innate immune responses and inflammatory-response-associated pathways were activated [38,43].

Virulent genotype VII strains induce profound IFN responses. Compared with the F48E9 strain, the genotype VIId GD strain induced a fiercely robust IFN-γ expression in the spleen at 48 h, but other cytokines such as IL-6, AvBD2, and AvBD3 did not significantly change. As for other tissues, in the lung and Harderian glands, IFN-γ, IL-6, iNOS, AvBDs, TLRs, and MHCII were expressed at significantly higher levels in chickens challenged with the GD strain rather than with the viruses of the F48E9 strain group [44]. Additionally, the genotype VIId go/CH/LHLJ/1/06 strain is also an efficient stimulator of IFN-γ in geese, which is accompanied by an intense innate immune response [45]. In another study, the genotype VIId virulent NDV/chicken/Egypt/1/2015 strain fiercely stimulated chIFNα in the spleen of broilers within 24 h [46]. In comparison to the VII Duck/CH/GD/SS/10 strain, type I IFN induction was minimal in CEFs and DEFs after IX Duck/CH/GD/NH/10 strain infection [47]. Infection with genotype VIId NDV strains (JS5/05 and JS3/05) also led to the hyperinduction of type I interferons (IFNs) (IFN-α and -β) and type II interferon (IFN-γ) in chicken splenocytes from 6 to 24 h [48]. It is of note that IFN expression levels are not necessarily decisive relative to the pathogenicity of the strains. Kang et al. reported that the virulent genotype VII Duck/CH/GD/SS/10 strain and genotype IX Duck/CH/GD/NH/10 strain induced significantly higher type I and II IFNs in CEFs than in DEFs, which correlates with the viral titer [47]. On the contrary, the 9a5b NDV strain infection leads to obvious clinical symptoms in chickens but not in ducks. In ducks, the IFN-β level in the lungs was significantly higher than that in chickens [42]. The expression level of IFNs is also cell type specific. For instance, when infected with the Herts/33 strain, primary chicken intestinal epithelial cells (IECs) can produce a greater IFN response than CEFs [49].

In general, only genotype VII strains tended to generate a robust IFN response. For strains of other genotypes, the IFN levels after NDV infection are not necessarily related to the virulence of the strains. These levels are highly variable based on viral strains, cell types, and animal species. Therefore, as many virulent strains do not induce IFN expression, the IFN response only partially explains the high pathogenicity of genotype VII strains.

Apart from IFNs, IL-6 is the most frequently upregulated cytokine after virulent NDV replication [47]. The IL-6 mRNA was significantly elevated in Herts/33-, genotype VII Duck/CH/GD/SS/10 strain- and genotype IX Duck/CH/GD/NH/10 strain-infected CEFs and DEFs [47,50]. Genotype VII NDV strain IBS002 and genotype VIII NDV strain AF2240 also significantly stimulated IL-6 expression in the spleen of SPF chickens [40]. Meanwhile, the geese-origin genotype VII virulent go/CH/LHLJ/1/06 strain did not stimulate IL-6 expression in geese [45]. The genotype VII virulent GD strain elevated IL-6 mRNA levels in the lung and Harderian gland of one-day-old SPF chickens at 48 h after infection [44]. However, the velogenic strain F48E9 only increased IL-6 expression slightly both in the CEFs and BF of SPF chickens, and the lentogenic LaSota strain did not alter IL-6 levels [43,47,50].

## 4. Receptors for NDV PAMP

The antiviral process starts with the recognition of viral invasion. The hosts react to NDV infection via multiple receptors, which could be roughly classified as toll-like receptors (TLRs), retinoic-acid-inducible gene-I (RIG-I)-like receptors (RLRs), and other receptors. Their role is to monitor the presence of viral molecules and initiate the inflammatory response and antiviral immune signaling pathways, therefore protecting the hosts.

TLRs are located at the surface or in the endosome of cells, recognizing the pathogen-associated molecular patterns (PAMPs) of the viruses or bacteria and triggering the activation of intracellular transcription factors and the expression of innate antiviral genes [51]. They are also crucial detectors and mediators during NDV infection. About ten TLRs were found to be present in avian species, including TLR2, TLR3, TLR4, TLR5, TLR7, and TLR21 [52]. Among them, TLR3 and TLR7 are important detectors in the endosome for detecting NDV and initiating innate pro-inflammatory responses [53]. NDV infection stimulates TLR3 and TLR7 expression in human, mouse, chicken, and duck cells, and when NDV invasion was disrupted with sialidase, TLR3 and TLR7 expression was inhibited in CEFs [47,54,55,56]. It was further detected that NDV-derived dsRNA colocalizes with TLR3 in subcellular structures, and the activation of TLR3 and TLR7 is associated with the NF-κB pathway of innate immune responses, proving the monitoring role of the TLRs against NDV infection [54]. Apart from TLR3 and TLR7, the overexpression of duck TLR5 in DF-1 and HeLa cells also initiated the NF-κB pathway and upregulated IL-6 promoter activities [57]. In geese, TLR1, TLR3, TLR5, TLR7, and TLR15 expressions were all upregulated after NDV genotype VIId strain infection, suggesting that these TLRs may also participate in anti-NDV responses, which needs further exploration [45].

RLRs are a group of key sensors in the cytoplasm against virus infection, triggering the transcriptional induction of type I interferons and other genes involved in the antiviral response. A number of RNA viruses, including SARS-CoV-2, influenza virus, and flavivirus, were reported to be recognized by RLRs [58,59,60]. RIG-I, MDA5, and LGP2 are homologous and comprise the RLR family [61]. RIG-I recognizes viral RNAs and depends on its N-terminal caspase recruitment domain (CARD) and C-terminal RNA helicase domain [62]. In mammals and waterfowls, RIG-I is one of the crucial molecules that protect cells and resist NDV infection [63,64]. RIG-I targets the triphosphorylated terminus of blunt-ended viral RNA duplexes. In mouse and human cells, NDV RNA is recognized by RIG-I in the cytoplasm, triggering a subsequent antiviral signal cascade during early infection [65]. The presence of RIG-I is highly related to the resistance ability relative to NDV. Wilden et al. found that, compared with macrophage-derived RAW tumor cells, the expression level of RIG-I was much higher in primary murine macrophages, which are more resistant to NDV infections than in RAW cells [66]. In addition, RIG-I is also absent in chickens, which is presumably attributable to their higher susceptibility to NDV compared with waterfowls such as ducks and geese [67,68]. Instead of RIG-I, NDV and other RNA viruses are recognized by the other two RLR family members, MDA5 and LGP2, which preserve antiviral competence in chickens [69,70,71,72,73]. The infection of the virulent genotype VIId NDV strain was accompanied by massive MDA5 expression in the oviducts of egg-laying hens and chicken bone-marrow-derived dendritic cells in the early infection stage [74,75]. The increased MDA5 transcriptive level was also reported in ducks, especially in lung and thymus tissues [68]. On top of this, chicken LGP2 and MDA5 can work together or independently to trigger IFN production. Chicken LGP2 and MDA5 possess special positively selected sites (PSSs) at the DECH helicase domain of MDA5 and the RD domain of chLGP2. The mutants are associated with high affinity relative to NDV RNA and chicken STING [69].

Apart from TLRs and RLRs, several other receptors are also reported to potentially detect NDV. Protein kinase R (PKR) is activated by long double-stranded RNA (dsRNA) molecules. It is upregulated during NDV infections, initiating the eIF2α signaling cascade and suppressing NDV via cap-mediated eIF2α-dependent protein synthesis [76]. However, PKR deficiency does not alter IFN-β responses to NDV infection in mouse embryonic fibroblasts [77]. Cyclic-GMP-AMP synthase (cGAS) had a cytosolic DNA-sensing ability to initiate the STING signal pathway and stimulate IFN production [78]. Zhu et al. found that NDV-induced IL-8 transcript production significantly decreased in cGAS-knockout chicken cells, suggesting that cGAS may also play a role in NDV infection [79]. Asp-Glu-Ala-Asp (DEAD)-box helicase 1 (DDX1) is a member of the DEAD box helicase family and was originally considered to regulate type I IFN and inhibit virus replication [80,81]. Although direct interaction with NDV was not proven, Cheng et al. found that Asp-Glu-Ala-Asp (DEAD)-box helicase 1 (DDX1) could strongly bind to poly (I:C) and can inhibit NDV replication via IRF7-mediated IFN activation [82,83]. In addition, they found another DDX family member DDX3X as a potential sensor for viral RNA, proven by the knockdown of DDX3X, which increased NDV yields, and the overexpression of DDX3X, which increased IFN-β production. Further investigations indicated that DDX3X activates IFN-β via the chSTING–chIRF7–IFN-β signaling axis [84].

In general, host cells react to NDV infection via multiple sensors. Apart from the traditionally known TLRs (TLR3, TLR5, and TLR7) and RLRs (RIG-I, MDA5, and LGP2), cGAS and DDX family members (DDX1 and DDX3X) were also recently identified as potential sensors for detecting NDV RNA. These receptors initiate their specific signal pathways involving type I IFN and other antiviral cytokines. However, not all provide certain evidence with respect to binding to NDV-derived RNAs. Moreover, whether their roles during infection are competitive, antagonistic, or synergistic is unknown. Further investigations are needed to clarify the NDV’s pathogenic mechanism.

## 5. Mechanisms for NDV to Facilitate Its Replication

At a cellular level, the mechanisms for NDV to facilitate its replication can roughly be summarized to be an IFN-related mechanism, autophagy, and apoptosis. These processes are also deeply interrelated with each other in terms of regulating the NDV infection process.

### 5.1. IFN

IFNs are a group of cytokines that are crucial for antiviral immune responses, including type I IFNs (IFNα, β, ε, κ, ω, and others), type II IFN (IFN-γ) and type III IFNs (IFN-λ1, IFN-λ2, IFN-λ3, and IFN-λ4). They bind to cell surface receptors in order to initiate antiviral responses via the JAK/STAT pathway, activating the heterotrimeric complex ISGF3 formed from STAT1/STAT2 and IRF9. The expression of IFN is highly integrated relative to the anti-NDV process. High levels of IFNs always have an inhibitory effect on NDV replication. Susta et al. found that virulent-NDV-induced mortality was preceded by fiercely secreted IFN-γ. Thus, the function of IFN-γ was determined by inserting chicken IFN-γ into a virulent NDV ZJ1 strain. The results revealed a protective role against virulent NDV infection in vivo but not in vitro [85]. The treatment of CEFs with chicken IFN-γ elicited an antiviral environment composed of ISGs [43].

**Figure 1:**
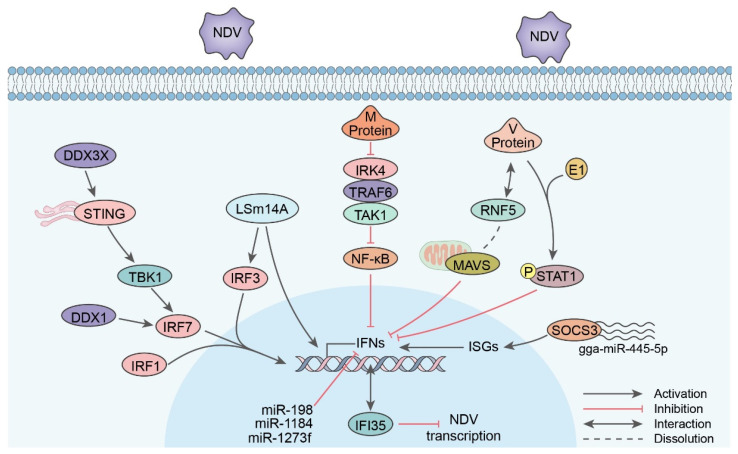
Signal pathway of IFN secretion caused by NDV infection.

Many antiviral-related proteins are involved in the anti-NDV processes. Proteins involving IFN secretion are prone to influence NDV replication (Figure 1). For instance, the overexpression of geese and chicken IRF1, IRF3, and IRF7 in chicken cells efficiently activates IFN-β, proinflammatory cytokines, and IFN-stimulated genes (ISGs), inhibiting the replication of NDV. This also applies to geese STING [86,87,88,89]. The chicken interferon (IFN)-stimulated 12-2 (ISG12(2)) gene is reported to attenuate the virulence of NDV via IFN secretion [90]. Jia et al. found that the distribution of interferon-induced protein-35 kDa (IFI35) mRNA in different tissues was positively related to NDV loads. The overexpression of IFI35 had an inhibitory effect on NDV replication, and it was positively involved with IFN modulators, suggesting that it may suppress NDV replication via the IFN pathway [91]. LSm14A is a member of the LSm family and binds to viral RNAs in the processing body (P-body), which mediates IRF3 activation and IFN induction [92]. As the chicken LSm14A mRNA level increases in NDV-infected tissues, it may also work as a detector to mediate innate immunity [93]. The Asp-Glu-Ala-Asp (DEAD)-box 3X-linked (DDX3X) polypeptide normally links to NLRP11 and NLRP3 to regulate IFN responses and inflammasome activation in mammals. In chickens, DDX3X was proven to interact with STING to stimulate IFN via the TBK1–IRF7 pathway, inhibiting NDV replication [94].

NDV also has its way to counteract the IFN secretion process, especially the virulent strains. Early research revealed that the NDV V protein can inhibit IFN-α. The carboxyl terminal domain of the V protein is responsible for limiting IFN’s ability to counteract viruses by degrading STAT1 [95]. In recent years, studies have found that the V protein targets MAVS degradation via the E3 ubiquitin ligase RING-finger protein 5 (RNF5), which leads to the inhibition of the downstream IFN-β pathway, thus favoring virus proliferation. This reveals a novel mechanism by which NDV evades the host’s innate immunity [15]. Apart from MAVS, the V protein can also degrade the phosphor-STAT1 protein via the ubiquitin E1-associated pathway to realize the IFN-α suppression effect [96]. Nan et al. further investigated the difference between the V protein from lentogenic and velogenic strains, showing that the lentogenic V protein inserted recombinant NDVs and induced lower IFN levels [13]. The IFN inhibition ability of the V protein is positively related to the virulence of viruses [97]. Another study showed that the V protein promotes the expression of cytokine signaling 3 (SOCS3) at the mRNA and protein levels via the MEK/ERK signaling pathway, thereby favoring viral replication [98]. Therefore, the ERK pathway can be studied as a potential antiviral target, which may provide new ideas for the development of antiviral strategies. The other non-structural protein W was also recently found to be related to the expression of IFN-β. To be specific, the W protein of the SG10 strain localized in the cytoplasm while that of the LaSota strain localized in the nucleus. The cytoplasm-localized W protein was associated with higher IFN-β expression at early infection stages, which inhibited NDV replication. The nuclear-localized W protein inhibited the production of IFN-β, thereby improving the replication, virulence, and pathogenicity of NDV [16]. Apart from V and W proteins, the N-terminal 180 amino acids (aa) of skeleton protein M also proved to antagonize NF-kB activation via the IRK4/TRAF6/TAK1/NF-kB signaling pathway [99]. However, in this study, the M protein did not directly interact with these proteins. Therefore, further investigations about how the M protein activates the signal cascades are needed.

MicroRNAs (miRNAs) are a group of short RNAs that are crucial for host–pathogen interactions. The cellular miRNA gga-miR-455-5p level decreased after NDV replication, which targets SOCS3 to upregulate type I IFNs and ISGs [100]. Several other miRNAs (miR-1273f, miR-1184, and miR-198) transmitted by exosomes also have a promotive effect on NDV replication via the inhibition of the IFN pathway [101].

### 5.2. Autophagy

Autophagy, a lysosomal-pathway-mediated degradation process, has an essential role in the regulation of innate and adaptive immune responses. Autophagy is highly involved in many viral infection processes, showing promotive abilities in the hepatitis C virus, dengue virus, and Zika virus [102,103,104]. NDV also triggers autophagy, which benefits its replication in both human and chicken cells.

To be specific, it was primarily found that NDV strain Beaudette C infection in U251 glioma cells at 10 MOI induces autophagy as early as 2 h. The accumulation of the LC3 protein and the conversion of LC3-I to LC3-II was evident due to the increased autophagosomes. The PI3K/Beclin-1 pathway was involved in NDV-triggered autophagy, which in turn promoted NDV replication [105]. Further research confirmed the NDV-associated autophagy in chicken cells and tissues. When infected with NDV Herts/33 strain at 1 MOI, autophagy was detected in CEF and DF-1 cells from 6 h to 36 h. In vivo experiments displayed the conversion of LC3-I to LC3-II in the heart, liver, spleen, lung, and kidney of infected animals, which is positively related to the virus titer. The inhibition of autophagy by wortmannin, but not rapamycin, suppresses the replication both in vivo and in vitro [106,107]. Similarly, Ren et al. found that, although the effect of rapamycin was different, and the NDV GM strain can also induce autophagy in CEFs and chickens [108].

The mechanism of NDV-induced autophagy was also investigated. The structural proteins P or NP could trigger autophagy via endoplasmic reticulum (ER) stress–related unfolded protein response (UPR) pathways in A549 cells [109]. This is in line with the result of Wang et al., showing that when the ER stress and autophagy were inhibited by the COX-2 protein, the NDV F48E9 strain’s replication was also suppressed in DF-1 cells [110]. Apart from NP and P proteins, F- and HN-protein-induced syncytia were also involved in autophagy fluxes via the activation of the AMPK–mTORC1–ULK1 pathway [111]. The influence of autophagy on NDV replication is not limited to the direct promotion of virus replication; it is also associated with the shift of metabolic mechanisms toward the benefit of the virus. NDV infection activates the glycolytic pathway to elevate glucose utilization efficiency. This is achieved by mitochondrial damage, elevated mitochondrial reactive oxygen species (mROS), and ETC dysfunction [112].

In the oncolytic virus NDV FMW strain, autophagy is also highly associated with NDV FMW-strain-induced apoptosis in lung cancer stem cell (CSC)–enriched lung cancer spheroids via the inhibition of the AKT/mTOR pathway [113]. In agreement with this, in lung cancer cells and melanoma cells, the NDV FMW strain was proven to induce immunogenic cell death mediated by autophagy-related genes [107,114]. In addition, another NDV strain, Hitcher B1, has been shown to exert oncolysis in cervical cancer cells via autophagy in a dose-dependent manner [115].

**Figure 2:**
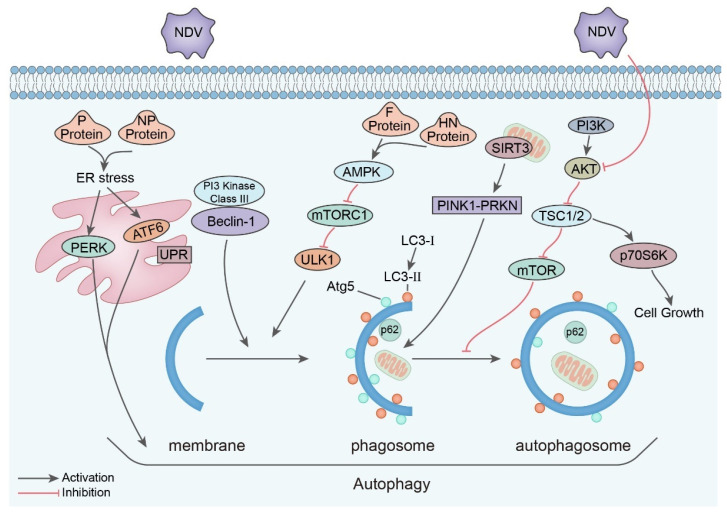
Autophagy in the pathogenesis of NDV.

In general, several NDV strains are able to induce autophagy to promote viral replication in human cancer cells and chicken cells, causing immunogenic cell death in cancer cells. The viral structural proteins, NP, P, HN, and F, are involved in this process via the UPR or AMPK–mTORC1–ULK1 pathways. On top of this, NDV also induces PINK1–PRKN-dependent mitophagy to increase glucose utilization for the benefit of viral production (Figure 2).

### 5.3. Apoptosis

Apoptosis is widely known as programmed cell death, showing a series of characteristic morphological changes along with several enzyme-dependent biochemical processes [116]. It is highly involved in NDV infection. NDV-induced apoptosis was first reported in 1994, when Lam et al. found that the NDV GB strain induced apoptosis in chicken peripheral blood lymphocytes (PBLs) and CEFs [117]. Since then, a number of NDV strains have been reported to induce apoptosis in both avian cells and human cancer cell lines. NDV entry into murine dendritic cells (DCs) induces extrinsic apoptosis and inhibits CD4^+^ cell proliferation [118]. When challenged with virulent NDV strains, apoptosis is detected in multiple organs, including the pancreas, resulting in disrupted exocrine and endocrine functions [30]. In the oviducts, severe tissue lesions and apoptotic bodies were detected, leading to oviduct dysfunction and a drop in egg production [74]. In lymphoid tissues, the amount of apoptosis was positively related to the severity of the clinical disease elicited by the strains [37]. As the infection duration lengthened, the secretion of pro-inflammatory cytokine expression, macrophage infiltration, and oxidative stress were also detected along with apoptosis in the BF [119].

**Figure 3:**
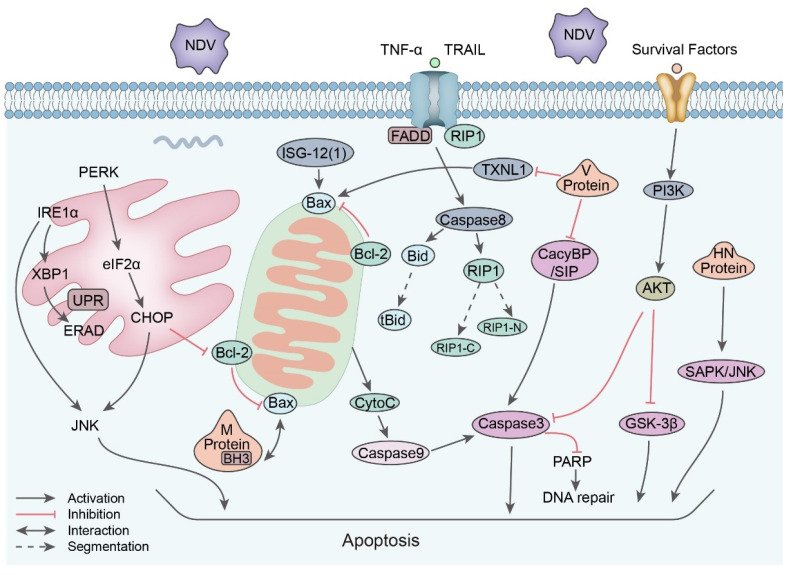
Apoptosis signaling pathway induced by NDV.

Apoptosis is an important mechanism of cells to resist infection by pathogens such as NDV. As a result of apoptosis, NDV replication is aborted in early infection stages and NDV release is promoted in late stages. To benefit its own replication, NDV employs multiple methods to postpone apoptosis (Figure 3). Ren et al. reported that the NDV activated the PI3K/Akt pathway in chicken cells immediately after infection, which suppressed premature apoptosis at the early stage of infection [120]. On the contrary, in the late stages of infection, both intrinsic and extrinsic apoptosis pathways will be triggered. The extrinsic pathway starts with the activation of NF-kB, followed by the secretion of TNF-α/TRAIL. It subsequently activates caspase 8 and cuts Bid into tBid, therefore initiating the intrinsic apoptosis process. Furthermore, caspase 8 also cleaves RIP1 to promote apoptosis, as the full length RIP1 functions against apoptosis [121]. As the ubiquitination status of RIP1 determines the apoptosis state, if it is also associated with NDV-induced apoptosis, it is worthy of further investigation [122]. Apart from the intrinsic and extrinsic apoptosis, Li et al. also reported the role of unfolded protein responses (UPRs) in NDV-induced apoptosis. It was found that all three branches of the UPR pathway (PERK–eIF2α, ATF6, and IRE1α) were active during late NDV infection. To be specific, the eIF2α–CHOP–BCl-2/JNK and IRE1α–XBP1/JNK pathways were triggered, which promoted apoptosis and benefited the replication of NDV [123]. Al-Shammari et al. found that NDV induces apoptosis both via the caspase-dependent (caspase 8 and caspase 9) and -independent (endonuclease G) pathways [124].

So, how do NDV particles manipulate these processes? Several research studies were published to answer the question. It was reported that structural protein M is necessary for apoptosis during infection. The NDV M protein directly interacted with the Bax protein via its BH3 domain. Compared with the full-length M protein, when the M protein was transfected with the deleted BH3-like region, it showed a five-fold decrease in apoptosis [125]. The V protein interacts with CacyBP/SIP to control viral propagation and cell death [126]. Musashi1 and NDV V proteins have a detectable interaction, and they may block apoptosis to inhibit the release of NDV [127]. On top of this, the HN protein is reported to induce apoptosis via the SAPK/JNK pathway [128]. The replication process of the viral gene may not be involved in NDV-mediated apoptosis, as the inactivated NDV is able to promote TRAIL-mediated apoptosis in tumors [129,130]. However, more remains to be investigated, such as how CacyBP/SIP and Musashi1 participate in the apoptosis process.

NDV normally induces the apoptosis of cancer cells in the late stages of infection, and this is extensively utilized in oncolytic research studies [131]. NDV treatments significantly decreased the viability of a TC-1 cell line and suppressed growth by inducing apoptotic cell death mediated by ROS production [132]. When apoptosis was inhibited via the treatment of z-VAD-FMK, NDV-mediated immunogenic cell death in prostate cancer cells was also attenuated [133]. Due to the importance of apoptosis in NDV replication, many apoptosis-related proteins influence NDV-mediated apoptosis. For instance, estrogen binds to estrogen receptor a (ERa) to induce apoptosis in breast cancer cells. The NDV-90 strain could further promote estrogen-mediated apoptosis in ERa-positive cells [130]. The classical oncology medication temozolomide can also act together with NDV to aggregate tumor cell apoptosis [134]. The TRAIL pathway is the main mechanism for mediating NDV-induced glioma cell apoptosis, and the co-administration of secreted TRAIL synergizes with NDV (MTH-68/H strain) to promote tumor cell death [135]. When integrated into NDV particle structures, TRAIL also has the same effect [136]. p53 is an important protein in apoptosis. The recombinant NDV expressing p53 (rNDV-p53) induced glioma cell apoptosis by upregulating apoptosis-related genes [137].

Many other antiviral proteins potentially influence apoptosis. Antiviral protein ISG12(1) could initiate apoptosis by redistributing Bax to hinder NDV replication [138]. Another immune-related protein, 2′-5′ oligoadenylate synthase-like (OASL) protein, also potentially exerts its antiviral ability via apoptosis, as the knockdown of OASL reduced the expression of apoptosis-related genes [139]. The TXNL1 protein induced apoptosis and inhibited NDV replication in DF-1 cells. Furthermore, Western blot and Q-PCR results suggested that TXNL1 induced cell apoptosis via a pathway involving Bcl-2\Bax and Caspase-3 [140]. The overexpression of Bcl leads to anti-apoptosis characteristics in the A549 cell line, and NDV selectively proliferates in these anti-apoptosis cells [131].

The biochemistry processes orchestrated in the body interact with each other; for instance, autophagy and apoptosis tend to work against each other during NDV infection. When autophagy is induced by rapamycin, apoptosis in the spleen and lung tissues will be diminished. In the meantime, apoptosis inhibitor ZVAD-FMK also promotes autophagy and viral replication [108]. Similarly, mitophagy also facilitates NDV replication by hindering apoptosis in lung cancer cells [141]. In addition, the tumor-selectivity of NDV is presumably due to the cumulative effect of type I and III in the tumor cells, which lead to a higher apoptotic effect [142]. All these facts confirm the complexity of the NDV infection mechanism, demanding more investigations.

### 5.4. Other Host Interaction Metabolism

The virus establishes intimate and complex interactions with host cells to counteract the antiviral response caused by the cell. This reinforces the fact that the virus coordinates the cellular antiviral response according to its own interests. Regulating the cell cycle is a fundamental cellular process that is important for cell proliferation, differentiation, and cellular homeostasis. Thus, disrupting homeostasis in the host cell cycle is a common strategy used by many viruses to create a cellular environment that is conducive to viral replication. An examination of various cyclin-regulatory proteins following NDV infection showed a significant decrease in cyclin D1 expression. In addition, NDV infections can also induce cell cycle arrest in the G0/G1 phase, thereby creating favorable conditions for viral replication [143]. NDV can select different types of cells for infection. NDV exhibits host cell tropism toward HeLa cells, which may be related to differences in receptor subtype expression patterns between different cell types. NDV can even autonomously select the cell cycle of infection. Studies have shown that NDV tends to infect cells in the S/G2 phase, during which cell proliferation favors viral replication, and enhanced viral replication leads to enhanced cell damage [144].

Paramyxoviruses inhibit antigen presentation in DCs via multiple mechanisms to enhance viral proliferation. NDV infection is able to induce dendritic cell (DC) phenotypic maturation and inhibit the proliferation of T cells via DC-mediated IL-10, thereby suppressing adaptive immunity [145]. The inhibition of DC antigen presentation may be a strategy used by NDV to disrupt the host’s adaptive immune response to prolong the persistent transmission of the virus. As foreign substances, viruses rely entirely on the host’s metabolic mechanisms and hijack host nutrients for viral replication. NDV can regulate the overall amino acid metabolism of infected cells to meet the needs of viral protein synthesis during viral replication and promote its own replication. NDV infections can induce a significant upregulation of the glutamate transporter gene, solute carrier family 1 member 3 (SLC1A3), which increases the uptake and transport of glutamate by a medium and cytoplasm in favor of viral replication [146].

The findings mentioned above represent the outcomes of research on the pathogenic mechanism of NDV; however, there is still more work to do. Studies on other paramyxoviruses have also been conducted in recent years, which may benefit future NDV studies. In eukaryotes, N^6^-methyladenosine (m^6^A) is the most common internal alteration of mRNA. Respiratory syncytial virus (RSV) replication, gene expression, and viral generation in HeLa and A549 cells were discovered to be favorably regulated by m^6^A methylation of the RSV genome, antigenome, and mRNA [147]. It suggests that m^6^A may be a novel target for the development of live-attenuated RSV vaccines and antiviral medications. Exosomes are vectors for the transfer of DNA and RNA viruses, and a growing body of research shows that they play significant stimulative and antiviral roles in many infection cycles. For instance, peste des petits ruminants virus (PPRV) can release viral components via exosomes, enabling PPRV to be transmitted from cell to cell [148]. NDV infection will change the secretion and the contents of exosomes, transferring viral NP protein and promoting NDV infection [149]. In the meantime, NDV could also benefit its replication through exporting NLRX1 mRNA to relieve the antiviral pressure on its survival [150]. However, the specific role of exosomes in NDV infection still needs further study. Secondary viral or bacterial infections are becoming increasingly prevalent, particularly in respiratory disorders. Recent studies have shown that complex interactions between the respiratory microbiome, host immune response, and viruses may have an impact on the pathogenesis and severity of RSV infection. RSV infection may cause damage to the lungs, making them more vulnerable to bacterial infections. During an RSV infection, the expression of some bacterial receptors is elevated, increasing bacterial susceptibility [151]. To provide theoretical support for future vaccine design as well as disease prevention and control, more study is required to better understand the processes of these interactions. A significant field of cell biology study is glycomics. N-glycosylation changes that are connected with viruses have many benefits for viral virulence and survival. Glycosylation of structural proteins associated with the Nipah virus (NiV), the Hendra virus (HeV), and the respiratory syncytial virus (RSV) has been demonstrated to affect viral invasion, viral replication, and syncytial development [152]. The processes by which viral proteins are glycosylated during viral infection and replication will guide the creation of particular antiviral treatments and vaccines.

## 6. Conclusions

The host and virus have long evolved to compete against each other, which is quite the case for NDV infections. The host cells employ a number of PAMPs, including the TLRs, RLRs, DDX family members, and cGAS, to initiate the antiviral IFN response via different pathways. The proteins belonging to these pathways are also potential anti-NDV candidates, such as IRF1, IRF3, IRF7, IFI35, LSm14A, and STING. Autophagy and apoptosis are normally considered to be cell defense mechanisms. They both have their specific roles in NDV infection, as autophagy promotes NDV replication and apoptosis inhibits viral replication at the early stage and facilitates it at late stages via different pathways. In the meantime, the virus is also able to take advantage of these reactions to benefit its own replication. In general, as an oncolytic virus and an important pathogen to avian species, NDV’s pathogenic mechanisms are crucial for the utilization of the virus and the development of the ND control strategy. Apart from the advances summarized here, there remains a lot to be explored in the future.

## Data Availability

No new data were created or analyzed in this study. Data sharing is not applicable to this article.
